# Befriending the Hostile Tumor Microenvironment in CAR T-Cell Therapy

**DOI:** 10.3389/fimmu.2020.618387

**Published:** 2021-02-10

**Authors:** Lorenzo Lindo, Lauren Hanna Wilkinson, Kevin Anthony Hay

**Affiliations:** ^1^ Terry Fox Laboratory, BC Cancer Research Institute, Vancouver, BC, Canada; ^2^ Department of Medicine, Faculty of Medicine, University of British Columbia, Vancouver, BC, Canada

**Keywords:** chimeric antigen receptor, immunotherapy, cytokine release syndrome, immune effector cell-associated neurotoxicity syndrome, immunosuppression, tumor microenvironment

## Abstract

T-cells genetically engineered to express a chimeric antigen receptor (CAR) have shown remarkable results in patients with B-cell malignancies, including B-cell acute lymphoblastic leukemia, diffuse large B-cell lymphoma, and mantle cell lymphoma, with some promising efficacy in patients with multiple myeloma. However, the efficacy of CAR T-cell therapy is still hampered by local immunosuppression and significant toxicities, notably cytokine release syndrome (CRS) and neurotoxicity. The tumor microenvironment (TME) has been identified to play a major role in preventing durable responses to immunotherapy in both solid and hematologic malignancies, with this role exaggerated in solid tumors. The TME comprises a diverse set of components, including a heterogeneous population of various cells and acellular elements that collectively contribute towards the interplay of pro-immune and immunosuppressive signaling. In particular, macrophages, myeloid-derived suppressor cells, regulatory T-cells, and cell-free factors such as cytokines are major contributors to local immunosuppression in the TME of patients treated with CAR T-cells. In order to create a more favorable niche for CAR T-cell function, armored CAR T-cells and other combinatorial approaches are being explored for potential improved outcomes compared to conventional CAR T-cell products. While these strategies may potentiate CAR T-cell function and efficacy, they may paradoxically increase the risk of adverse events due to increased pro-inflammatory signaling. Herein, we discuss the mechanisms by which the TME antagonizes CAR T-cells and how innovative immunotherapy strategies are being developed to address this roadblock. Furthermore, we offer perspective on how these novel approaches may affect the risk of adverse events, in order to identify ways to overcome these barriers and expand the clinical benefits of this treatment modality in patients with diverse cancers. Precise immunomodulation to allow for improved tumor control while simultaneously mitigating the toxicities seen with current generation CAR T-cells is integral for the future application of more effective CAR T-cells against other malignancies.

## Introduction

Genetically engineering T-cells to express a chimeric antigen receptor (CAR) has gathered momentum over the past decade, leading to the FDA approval of three anti-CD19 CAR T-cell products: tisagenlecleucel (Kymriah), axicabtagene ciloleucel (Yescarta), and brexucabtagene autoleucel (Tecartus) (1). These products are indicated for several CD19^+^ B-cell malignancies, including B-cell acute lymphoblastic leukemia (B-ALL), diffuse large B-cell lymphoma (DLBCL), and mantle cell lymphoma (MCL). These products offer new hope for disease control and possible long-term remission in cancer patients with no other therapeutic option. While these current US Food and Drug Administration (FDA)-approved CAR T-cells are only indicated for B-cell malignancies, many novel CAR T-cells targeting various antigens are being developed for other cancers, including BCMA for multiple myeloma (MM), EGFRvIII for glioblastoma multiforme (GBM), and MUC1* for breast cancer, among others (2).

**Figure 1:**
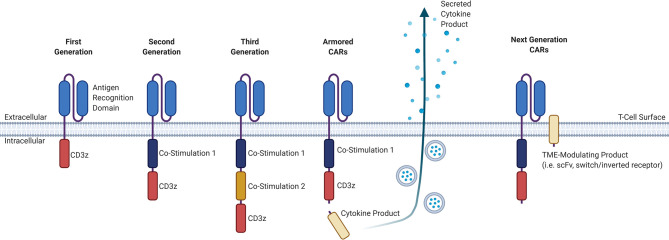
Evolution of the general structure of CARs from first-generation CARs to next-generation CARs. First-generation CARs contained only an antigen-recognition domain and a CD3ϛ T-cell signaling domain. Second-generation CARs added a co-stimulatory domain upstream of the CD3ϛ domain. Third-generation CARs have an added co-stimulatory domain. Armored CARs are a fourth-generation CAR that is based off of second-generation CARs and contain a secreted cytokine product that is either constitutively or inducibly expressed. Next-generation CARs will have a TME-modulating product, that may consist either of an scFv to sequester or deplete a certain factor in the TME, or a switch/inverted receptor to convert an adverse signal into a beneficial intracellular signal. CAR, chimeric antigen receptor; CD, cluster of differentiation; TME, tumor microenvironment; scFv, single-chain variable fragment.

CAR T-cell products that are currently commercially available involve the adoptive transfer of genetically-modified autologous peripheral blood T-cells, and although “off-the-shelf” allogeneic products are in development, these are only available in early phase clinical trials. The manufacture of autologous CAR T-cells involves the collection of T-cells from a patient via leukapheresis, lentiviral transduction of these collected T-cells with the CAR construct, expansion of these cells in a clinical laboratory, and infusion back into the patient, with a manufacturing timeline of approximately two to three weeks depending on the product (3). The CAR construct itself consists of an extracellular antigen-targeting domain and intracellular signaling domains, connected by a transmembrane linker, that collectively allows for non-MHC-restricted activation of T-cell effector functions upon binding of the CAR-expressing T-cell to its cognate antigen (3). While the extracellular antigen-targeting domain typically consists of a single-chain variable fragment (scFv) derived from an antibody, novel approaches for the antigen-targeting domain are being developed, including the use of ligand-receptor interactions to facilitate binding, such as the use of APRIL-based CARs for dual targeting of BCMA and TACI in MM (4, 5). Other examples of alternative antigen-targeting domains currently being developed for CAR T-cell therapy include single-domain antibodies termed “nanobodies”, as well as synthetic binding platforms such as Centyrin (6, 7). The intracellular signaling domains typically consists of the CD3-zeta (CD3ζ) T-cell signaling domain that may be combined with one (second generation) or more (third generation) co-stimulatory molecules, such as CD28 or 4-1BB. There are currently many other promising co-stimulatory molecules and different combinations of those co-stimulatory molecules being studied for their effect on CAR T-cells, including OX40, CD27, CD40, and ICOS (8). Newer fourth-generation CAR T-cells, also known as armored CARs or TRUCKS (T-cells redirected for antigen unrestricted cytokine-initiated killing), that secrete cytokines or produce transcription factors are being developed with a goal to improve activation efficiency, function, and survival. The different CAR generations are summarized in **Figure 1**.

While CAR T-cell therapy has yielded impressive therapeutic outcomes and significant anti-tumor activity in relapsed/refractory patients with no other therapeutic option, toxicities precipitated by this therapy have been identified. These toxicities, primarily cytokine release syndrome (CRS) and immune effector cell associated neurotoxicity syndrome (ICANS), occur following the activation and proliferation of CAR T-cells *in vivo*, resulting from a cascade of inflammatory cytokines precipitated by CAR T-cell activation (9). CRS occurs in 54% to 91% of patients after CD19 CAR T-cell therapy, with severe CRS occurring in 8.3% to 43%, depending on the type of CAR T-cell therapy product and grading system used (10–15). CRS generally presents 1 to 6 days post-CAR T-cell infusion, with fever as the hallmark sign, and is often accompanied by other non-specific flu-like symptoms, hypotension, and/or hypoxia. Patients who experience more severe CRS typically have earlier CRS onset and longer CRS duration after CAR T-cell infusion (16). In severe cases, other adverse events may occur, including capillary leak syndrome, vasodilatory shock, coagulopathy, and multiorgan failure, with a cytokine signature similar to macrophage activation syndrome/hemophagocytic lymphohistiocytosis (17–21).

ICANS occurs on average 5 to 7 days post-CAR T-cell infusion and can occur concurrently with or separately from CRS. However, higher grade neurotoxicity has been associated with higher grade CRS, suggesting that their respective risk factors overlap (20). ICANS can manifest as a broad range of symptoms including headache, encephalopathy or delirium, movement disturbances, apraxia, reduced attention, decreased level of consciousness and speech impairment (20–22). While infrequent, severe cases of focal neurological deficits, seizures, and in very severe cases, coma, intracranial hemorrhage, and acute cerebral edema have been reported (20–22). Depending on disease type and CAR T-cell product used, ICANS occurs in 32-64% of patients, with severe symptoms occurring in 11–42% of patients (11, 14, 15, 18, 20, 21, 23–25). Patients with severe ICANS show signs of endothelial dysfunction including vascular leak, endothelial activation and increased blood brain barrier (BBB) permeability, which allow systemic cytokines to accumulate in cerebrospinal fluid, thus exacerbating neurotoxicity (21). Additionally, brain mural cells which stabilize the BBB have been seen to express CD19, suggesting, at least with CD19 CAR T-cells, a possible “on-target, off-tumor” effect that may worsen disruption of the BBB (26).

Early studies in CAR T-cell therapy identified that cytokine blockade with tocilizumab, an anti-interleukin (IL)-6 receptor (IL-6R) monoclonal antibody, was highly effective at treating CRS without compromising efficacy, leading to FDA approval of tocilizumab for this indication (11, 16, 27). In more severe cases of CRS, corticosteroids as a broad immunosuppressive approach have also been effective, and are particularly useful for the treatment of ICANS, for which tocilizumab appears to have a limited role (11, 16, 18). Dexamethasone is one of the corticosteroids of choice for the management of severe CRS, due to its effective penetration of the blood-brain barrier which may abrogate some of the symptoms of ICANS (28, 29). Early use of corticosteroids does not appear to compromise the CAR T-cell anti-tumor responses; however, concerns still exist that high cumulative doses may impact efficacy (30).

Despite improvements in our management of these toxicities, further optimization is needed, particularly for the treatment of ICANS. Alternatives to IL-6R blockade and corticosteroids are currently being studied for their use in the management of CRS and ICANS, including siltuximab, an anti-IL-6 monoclonal antibody that is used clinically for Castleman’s disease, and anakinra, a recombinant IL-1RA protein used clinically for rheumatoid arthritis and that can block IL-1, a cytokine that has been implicated in CRS and ICANS pathogenesis (22, 31, 32). Several recent studies have looked at alternative CAR T-cell strategies to improve the safety profile, such as engineering CAR T-cells with suicide genes, ON- and OFF- switches, AND/OR logic gating, or various inhibitory domains (33, 34). However, these strategies directly limit CAR T-cell function. Further research into the pathophysiology and in particular the role of the microenvironment is needed to uncover novel pathways that can be targeted to mitigate severe toxicities in CAR T-cell therapy without limiting CAR T-cell effector function.

CAR T-cells are “living drugs” that proliferate and expand in the recipient in response to signals provided *in vivo*, such as cognate antigen availability, and are targets of many of the same local and systemic regulatory signaling pathways as their endogenous T-cell counterparts. Consequently, CAR T-cells are susceptible to the myriad of homeostatic mechanisms that regulate immune function in the host. Locally, the tumor microenvironment (TME) provides strong immunosuppressive and immunomodulatory signals through cell-cell interactions and secreted factors. This has led many groups to explore the individual roles of the different cell types and cell-free factors contained within the heterogeneous population of cells present in the TME to identify specific signals and cell types implicated in mediating CAR T-cell inhibitory signaling.

The advent of novel CAR T-cell strategies to overcome these immunosuppressive signals offers renewed hope for CAR T-cells as possible curative strategies in many cancers. CAR T-cells that have been engineered to inducibly or constitutively secrete CAR T-cell-potentiating molecules are being studied to improve efficacy and persistence. Often, the secreted agent selected is based on perceived immunosuppressive signaling in the TME, and thus these cytokines have been chosen to create an immune-supporting milieu. However, while stimulating pro-immune signaling may improve the function of CAR T-cells, they may paradoxically and unintentionally potentiate some of the adverse events associated with CAR T-cells, which have been largely attributed to excessive systemic pro-inflammatory signaling leading to CRS and ICANS. Herein we review the mechanisms by which the TME impacts both toxicity and efficacy, and how newer methods to target the TME may impact these outcomes.

## The Role of the TME in CAR T-Cell Therapy

### How the TME Impacts CRS/ICANS

Upon activation following recognition of and binding to their cognate antigen, CAR T-cells proliferate, expand, and secrete cytokines into the tumor milieu in order to create a proinflammatory environment and activate nearby immune cells. Data from pre-clinical and clinical studies suggests that the source of the majority of these supraphysiologic cytokine levels seen in CRS is not the CAR T-cells themselves; rather, it is the activation of these surrounding cells that creates the cascade of cytokines leading to clinical CRS. In particular, macrophages and endothelial cells have been found to play a key role in the development and pathogenesis of CRS, through the mediation of core cytokines, cell-cell interactions with CAR T-cells, as well as their involvement in self-amplified catecholamine loops.

#### Macrophages

Macrophages play a critical role in both the development and progression of CRS and ICANS following CAR T-cell therapy. Using two different murine models of CRS, Giavridis et al. and Norelli et al. both demonstrated the key role of the monocyte-macrophage lineage in CRS pathogenesis (35, 36). Using SCID/beige mice grafted with an intraperitoneal high tumor burden to induce CRS, Giavridis et al. observed increases in F4/80^int-lo^Ly6C^int-hi^ macrophages, which, following administration of mCD40L CD19 CAR T-cells engineered to further engage macrophages, led to markedly increased macrophage numbers, CRS symptoms, and mortality. IL-6 was predominantly produced by these macrophages, and blockade with anti-murine IL-6R antibody or with anakinra prevented CRS. Of interest, IL-1 release proceeded IL-6 production by hours, suggesting IL-1 lies further up in the cascade that leads to IL-6 release. In a humanized mouse model, Norelli et al. demonstrated that human monocytes were the predominant source of IL-1 and IL-6. Blockade with tocilizumab prevented CRS; however, blockade with anakinra prevented both CRS and ICANS. Both IL-1 and IL-6 are inducers of inducible nitric oxide synthase (iNOS), and upon their activation, macrophages produce excess nitric oxide (NO), leading to increased hypotension and vasodilation, two common symptoms associated with CRS (35). Treatment of mice with iNOS inhibitors L-NIL and 1400W both decreased mortality and reduced toxicity in tumor-bearing mice treated with CD19-directed CAR T-cells (35). Blockade of either IL-1 or IL-6 was sufficient to reduce iNOS^+^ macrophage abundance and reduce toxicity. These data have led to clinical trials of anakinra for CRS (NCT04359784, NCT04148430, NCT04150913).

One recently proposed mechanism by which macrophages are activated in the CRS cascade is a highly inflammatory form of programmed cell death, pyroptosis. Liu et al. recently demonstrated that CD19 CAR T-cells, upon exposure to B cell leukemia, release excessive granzyme B, which enters the target tumor cells leading to activation of caspase 3 (37). Activated caspase 3 subsequently cleaves gasdermin E (GSDME) generating its active form (38). Human B leukemic cells ubiquitously express high amounts of GSDME, which, upon activation, form oligomers that insert into cell membranes, causing pyroptosis, evidenced by swollen dying cells with blebs in the plasma membrane and high levels of lactate dehydrogenase (LDH) (37, 39). The knockdown of perforin, GSMDE blockade, as well as caspase 3 and granzyme B inhibitors were all shown to inhibit pyroptosis in tumor cells. Once tumor cell pyroptosis occurs, damage-associated molecular pattern molecules (DAMPs) are released, which leads to the activation of caspase 1 and GSDMD in macrophages and production of CRS-related cytokines (39, 40). Macrophages treated with the pyroptotic supernatants from CD19 CAR T-cells co-cultured with various leukemic cell lines produced excessive amounts of IL-6 and IL-1β (37). Using the CRS murine model previously described by Giavridis et al., Liu and colleagues demonstrated that knocking out GSDME, depleting macrophages, or inhibiting caspase 1 eliminated CRS (37).

Another proposed mechanism by which CAR T-cells activate macrophages is direct activation through secretion of granulocyte-macrophage colony-stimulating factor (GM-CSF). In the ZUMA-1 trial, high levels of GM-CSF were found to be associated with severe neurotoxicity (21). Using CAR T-cell/macrophage trans-well assays, Sachdeva et al. identified that the major source of GM-CSF is the CAR T-cells, and confirmed macrophages as the source of IL-6 and monocyte chemoattractant protein-1 (MCP-1) (41). TALEN-mediated genetic inactivation of GM-CSF in the CAR T-cells subsequently abolished the CRS cytokine production by macrophages (41). Supporting these data, in a patient-derived xenograft mouse model Sterner et al. showed that CAR T-cells combined with lenzilumab, a GM-CSF neutralizing antibody, or CRISPR-Cas9 generated GM-CSF^KO^ CAR T-cells reduced CRS and neuroinflammation (42). Furthermore, neutralization of GM-CSF appeared to the enhance anti-tumor activity of CAR T-cells, suggesting that inhibition of GM-CSF may in fact both reduce toxicity and enhance responses.

In addition to the key role of macrophage-produced cytokines in CRS, macrophage release of catecholamines can also mediate CAR T-cell therapy-related toxicities. An association was seen between patients with high peak levels of noradrenaline (NAD) following administration of standard of care axicabtagene ciloleucel (axi-cel) and grade ≥3 CRS, suggesting a link between catecholamine production and clinical toxicity (43). In a model by Staedtke et al., CRS was accompanied by a catecholamine surge and inhibition of catecholamine synthesis protected mice from fatal CRS (44). Using peritoneal macrophages from mice with selective deletion of the *Th* gene in LysM^+^ myeloid cells, they confirmed that the production of catecholamines by macrophages drives the inflammatory response, with Th-deleted macrophages showing decreased catecholamine production and CRS (44). Mice treated with methyltyrosine (MTP) and atrial natriuretic peptide (ANP) prior to CD19-CAR T-cell therapy demonstrated improved survival, as these molecules abrogated the increase in catecholamine levels and protected against CRS-associated mortality, while having little effect on anti-tumor efficacy of the CAR T-cells (44). These data suggest a mechanism by which T-cell-activated macrophages secrete catecholamines that act through adrenergic receptors, which can then promote a positive-feedback loop that can upregulate the inflammatory response, leading to CRS.

In addition to tumor associated macrophages, Deng et al. found that grade 3 to 4 ICANS was significantly associated with a rare monocyte-like population present in patients treated with Yescarta (axicabtagene ciloleucel) (45). They named this cell population ICANS-associated cells (IACs) and their presence is associated with lower detectable CAR expression and increased expression of genes implicated in ICANS pathophysiology, particularly IL-1β (45). Using their scRNA-seq dataset, they identified IAC signature genes and noted that this signature was significantly elevated in cells of the myeloid lineage. However, these IACs do not express canonical monocyte markers, such as CD14 or CD16, and therefore cannot be definitively ascribed to the myeloid lineage. As these IACs were found in all patients with grade 3-4 ICANS in this study, the presence and level of these IACs may offer some predictive benefit and thus further study may be warranted in this newly-identified cell population.

#### Endothelial Cells

A known hallmark of severe CRS is the activation of endothelial cells. Elevated levels of angiopoietin-2 (Ang-2) and von Willebrand factor (vWF), which are released from Weibel-Palade bodies upon endothelial activation, have been associated with both severe CRS and ICANS (16). Ang-2 and angiopoietin‐1 (Ang-1) are antagonistic ligands of the Tie-2 receptor, and under normal conditions the concentration of Ang-1 exceeds that of Ang-2 promoting endothelial integrity and stability. Excessive Ang-2, seen by a high Ang-2:Ang-1 ratio leads to endothelial permeability, induction of adhesion molecules on endothelial cells, and migration of cells from the vasculature, creating a proinflammatory state (16, 46–49). Under normal conditions, vWF stabilizes the adhesion of platelets at sites of vascular injury; however, during inflammation excess vWF multimers self-associate on the endothelium, contributing to thrombosis and increased vascular permeability (50).

*Trans*-signaling of IL-6 by endothelial cells has been associated with CRS due to other etiologies such as sepsis, acute respiratory distress syndrome (ARDS), and severe burn injuries (51–53). IL-6 *trans*-signaling occurs when soluble IL-6 receptor (sIL-6R) interacts with IL-6 in the blood, then binds to gp130 on cells that classically lack the IL-6R, such as endothelial cells (53). Using a sepsis model, engagement of Toll-like receptor 4 (TLR4) on endothelial cells via lipopolysaccharide (LPS) produces excessive IL-6, which in turn interacts with sIL-6R and gp130 to create a positive feedback loop of more IL-6 production as well as a variety of other proinflammatory molecules, such as plasminogen activator inhibitor 1 (PAI-1) (53).

**Figure 2:**
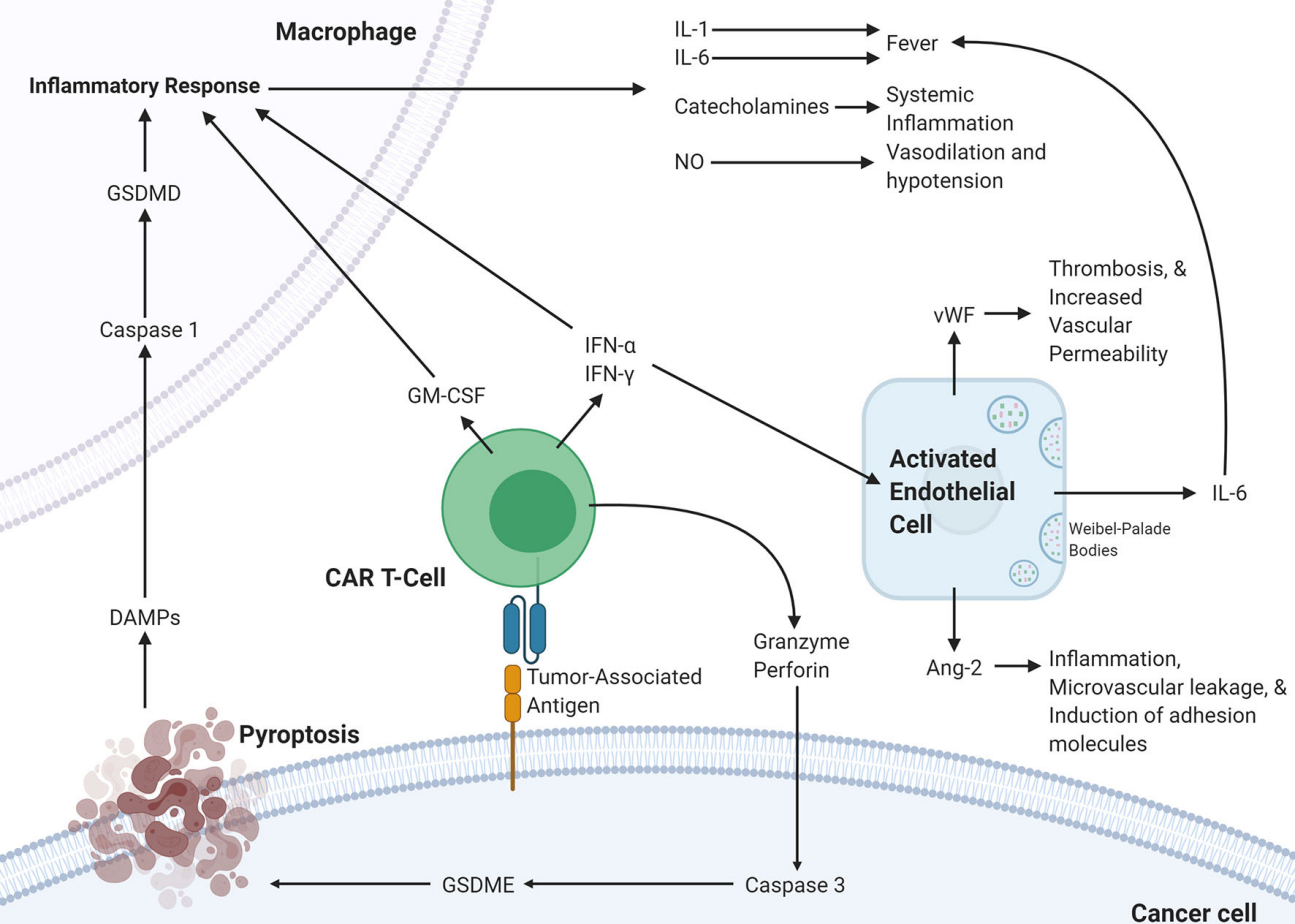
Proposed pathophysiology of CRS/ICANS. Activation of CAR T-cells upon encountering its cognate antigen on a target cell results in the CAR T-cell release of granzyme, perforin, IFN-α, IFN-γ, and GM-CSF. These cytokines trigger several events on the surrounding cells. In the cancer cell, granzyme results in the activation of caspase-3 which activates gasdermin E that leads to pyroptosis of the cancer cell. The subsequently released DAMPs bind to receptors on macrophages leading to caspase-1 activation which activates gasdermin D that results in activation of an inflammatory response. The released IL-1, IL-6, catecholamines, and NO lead to several different effects. This inflammatory program can also be promoted by GM-CSF, IFN-α, and IFN-γ released by CAR T-cells. IFN-α and IFN-γ can also activate endothelial cells leading to the release of IL-6, vWF, and Ang-2, which lead to several changes affecting the vasculature. CAR, chimeric antigen receptor; Ang-2, Angiopoietin 2; IFN-γ, interferon-gamma; IFN-a, interferon-alpha; vWF, von Willebrand factor; GM-CSF, Granulocyte-macrophage colony-stimulating factor; GSDME; Gasdermin E; GSDMD, Gasdermin D; DAMPs, Damage-associated molecular patterns; NO, nitric oxide.

The various mechanistic processes underlying CRS and ICANS have been summarized in **Figure 2**. It is clear that the pathophysiology underlying CRS and ICANS is complex and involves an interplay by many factors that are still under investigation. This evidence has implicated the TME in the pathophysiology of the adverse events associated with CAR T-cell therapy and several studies have shown that blocking certain pro-inflammatory pathways in the TME can abrogate CRS and ICANS. It appears that blocking these TME-related pathways does not hamper CAR T-cell efficacy; however, any further discussion of targetable pathways to decrease toxicity warrants an overview of the effect of the TME on efficacy.

### How the TME Impacts Efficacy

The TME is a significant barrier to durable remissions after immunotherapy in patients with hematological and solid tumors. In the setting of CAR T-cell therapy, this heterogeneous population of cells and factors provides a strong immunosuppressive signal. Studies exploring the effects of the TME on various immunotherapeutic strategies is a rapidly growing field of research, and studies are beginning to shed light on the specific mechanisms underlying the interaction between the CAR T-cells and specific factors in the TME.

#### Tumor-Associated Macrophages

Macrophages are classically divided into M1 and M2 macrophages based on their activation status; however, recent data shows that macrophages do not fit nicely into two distinct groups (54). Rather, the phenotypic plasticity of macrophages is better described as a spectrum from pro- to anti-inflammatory, with the former associated with anti-tumor and anti-infection roles and the latter associated with wound-healing and tumor-supporting roles (55, 56). In addition to their roles as phagocytes and as antigen-presenting cells, macrophages secrete various factors that contribute either to their immune-supporting or immune-inhibiting functions. In the context of the TME, macrophages are typically referred to as tumor-associated macrophages (TAMs). Multiple studies of TAMs in varying of malignancies have associated an increased abundance of TAMs with poorer outcomes (57, 58).

A phase I clinical trial (NCT03355859) of anti-CD19 CAR T-cells for patients with relapsed/refractory B-cell non-Hodgkin’s lymphoma (B-NHL) found that increased TAM infiltration was negatively associated with remission status (59). They quantified the expression of CD68, a general marker for cells of the macrophage lineage, and the expression of CD163, a marker associated with the M2 alternatively-activated and anti-inflammatory macrophages. Poorer outcomes were associated with significantly increased infiltration of both CD68^+^ and CD163^+^ macrophages. Furthermore, they showed that co-culture of M2 macrophages significantly decreased CD4^+^ and CD8^+^ T-cell proliferation compared to T-cells alone.

#### Myeloid-Derived Suppressor Cells

Myeloid-derived suppressor cells (MDSCs) are a heterogeneous population of cells of the myeloid lineage that consists of myeloid progenitors, including immature macrophages, immature granulocytes, and immature dendritic cells (DCs), all of which are involved in several immune processes (60). Whereas in healthy individuals, immature myeloid cells in the bone marrow can differentiate into mature granulocytes, macrophages, or DCs, in pathological conditions, MDSCs have been hypothesized to arise from a block or interruption in this differentiation scheme into mature myeloid cells, thereby resulting in an expansion of the MDSC population. These cells are characterized by their ability to suppress both innate and adaptive responses, and have been implicated in cancer, infection, and various inflammatory diseases (61). They constitute a unique component of the human system that has a role in regulating these immune responses in both healthy individuals and in various disease contexts. Specifically, MDSCs have been shown to accumulate in tumors in response to various cytokines and growth factors, to have upregulated expression of known immunosuppressive factors, and to act as suppressors of T-cell function (62).

Elevated levels of circulating MDSCs has been shown to be a poor prognostic indicator in a meta-analysis of patients with solid tumors (63) and is associated with minimal-residual disease (MRD)-positivity in leukemia as well as in other hematologic malignancies (64). It is likely that local and systemic immunosuppression by MDSCs leads to these associations, and thus MDSCs may conceivably interact with CAR T-cells. Indeed, studies have shown that MDSCs directly inhibited GD2-directed CAR T-cells in a mouse model for sarcoma (65). The authors of this study further showed that eradication of MDSCs using all-*trans*-retinoic acid (ATRA) was sufficient to diminish the suppressive capacity of granulocytic MDSCs and that combination of GD2-directed CAR T-cells with ATRA led to improved anti-tumor function compared to CAR T-cells alone (65). Depletion of MDSCs *in vitro* using Gemtuzumab ozogamicin (Mylotarg), an antibody-drug conjugate targeting CD33^+^, which is found on MDSCs (66), led to enhanced CAR T-cell function in various tumor models treated with CAR T-cells targeting either GD2, mesothelin, or EGFRvIII (67).

Studies have begun to elucidate the mechanisms underlying MDSC-mediated immunosuppression of T-cells in various tumor models. MDSCs are commonly trafficked to tumor cells and accumulate in the TME via chemokines and cytokines, including S100A8/A9, exosomal CD47, and IL-17 (68). A study of the TME in acute myelogenous leukemia (AML) has shown that there is contact-mediated effects between MDSCs and T-cells and the effects of this interaction include reduced T-cell proliferation and a switch from a Th1 to a Th2 phenotype (69). Interestingly, there seems to be differential effects of MDSCs on various subsets of T-cells. S100A9 knockout mice, which are deficient in their ability to accumulate MDSCs in tumor-bearing hosts, showed significantly reduced MDSC accumulation in the bone marrow along with significant accumulation of tumor-specific CD8^+^ cells, suggesting a possible CD8^+^-specific effect of MDSCs (70). Adding to the complexity, it has been shown that there are different subsets of MDSCs and that while these different MDSC subsets share in their ability to suppress antigen-specific T-cell responses, the required signaling pathways and factors differed among subsets (71). It was also unexpectedly found that some MDSC subsets can actually stimulate interferon-gamma (IFN-γ) production by CD8^+^ cells, which illustrates *some* potentially immune-supporting functions of MDSCs (72). It is clear that further studies should be conducted to characterize the different subsets of MDSCs present in the TME and how they interact with T-cells to uncover potential targets for intervention to shift the TME towards a more favorable environment.

The expansion of MDSCs in the TME in a model of liver cancer has been shown to be dependent on tumor-derived GM-CSF which promoted STAT3-mediated induction of PD-L1 on MDSCs leading to immunosuppression of CAR T-cells via direct engagement of PD-1 on CAR T-cells (73). The efficacy and function of CAR T-cells in this model was rescued by MDSC depletion, GM-CSF neutralization, or PD-L1 blockade (73). These findings suggest a potential combinatorial CAR T-cell therapy involving neutralizing antibodies targeting the GrI^+^ MDSCs themselves or targeting soluble factors involved in MDSC-mediated immunosuppression. Indeed, such an approach that targets the MDSCs in the TME to create a more favorable immune environment has been studied, such as IL-12 to reduce the T-cell-inhibiting functions of MDSCs while simultaneously activating innate myeloid cells, such as dendritic cells and macrophages (74). Recently, V-domain Ig suppressor of T-cell activation (VISTA), a known negative regulator of immune responses, was found to be highly expressed on MDSCs in the peripheral blood of AML patients (75). The authors showed that siRNA-mediated knockdown of VISTA abrogated MDSC-mediated inhibition of cytotoxic T-cells in AML, which suggests a likely mechanism of MDSC-mediated inhibition of CAR T-cells. In a nitroproteomic study, researchers found that lymphocyte-specific protein tyrosine kinase (LCK), a key factor involved in T-cell activation, is nitrated and subsequently inactivated by MDSC-derived reactive nitrogen species (RNS) in a mouse model of treatment-resistant prostate cancer (76). They showed that LCK nitration by MDSCs leads to diminished T-cell activation, reduced IL-2 production, and reduced T-cell proliferation. They further showed that in their mouse model of immune checkpoint inhibitor (ICB)-resistant prostate cancer that had diminished T-cell responses when treated with anti-PD-1 or anti-CTLA4 monoclonal antibodies, concomitant administration with an agent that neutralizes RNS led to strong tumor clearance, thereby supporting a rationale for using RNS-neutralizing compounds to overcome MDSC-mediated inhibition of T-cell responses.

Generally, it may be postulated that MDSCs cells may be involved in a cooperative interaction with macrophages due to their effects on macrophage cytokine production. MDSCs have been previously described to support tumor growth in a mammary carcinoma mouse model by MDSC-derived IL-10 production and by MDSC-mediated inhibition of macrophage IL-12 production (77), two cytokines involved in the regulation of T-cells. Further studies should aim to identify possible cooperative interactions between these two myeloid populations to determine how they may affect CAR T-cell function.

#### Regulatory T-Cells

Regulatory T-cells (Tregs) are a specialized subset of CD4^+^ T-cells that are distinct from the conventional CD4^+^ T helper (Th) cell lineage, characterized by their ability to suppress immunity to prevent any potentially deleterious effects of Th cells (78, 79). These effects are typically part of the normal immune homeostatic response to prevent over-activation of the immune system. However, in malignancy, Tregs can be prohibitive of immune-mediated anti-tumor responses. Increased circulating or tumor-infiltrating Tregs is associated with poor patient survival among several cancers including breast, melanoma, and lung, and has been reviewed elsewhere (80, 81). In a study of blinatumomab, a bispecific CD3/CD19 T-cell engager, responders were found to have significantly less Tregs compared to non-responders (82). Furthermore, they showed that depletion of Tregs *in vitro* using CD39-magnetic bead separation on frozen patient samples led to improved proliferation of effector T-cells, and that re-introduction of Tregs restored inhibition of proliferation in a dose-dependent manner. Similarly, in an *in vivo* model of AML, transient depletion of Tregs using IL-2-diphtheria toxin (IL-2DT) resulted in reduced AML tumor burden and improved the proliferation of adoptively-transferred tumor-reactive cytotoxic T-lymphocytes (CTLs) (83). Using a co-culture model that contained EBV-reactive CTLs and Tregs isolated from patients with Hodgkin’s lymphoma or healthy donors, administration of IL-15 promoted proliferation and effector functions of CTLs (84). They further showed that while IL-15 did not reverse or block Tregs, IL-15 was sufficient to favor the proliferation of CTLs and effector T-cells versus Tregs. Taken together, these findings suggest that administration of pro-inflammatory substances to counter the immunosuppressive functions of Tregs or depletion of Tregs can lead to improved function of effector T-cells.

Certain modifications to CAR T-cells have also been shown to have specific effects on their interactions with Tregs. Consistent with the heavily documented finding that CD28-containing CAR T-cells elicit stronger anti-tumor responses with increased cytokine release *in vitro* and *in vivo* (8, 85–92), CAR T-cells incorporating both CD28 and CD3 co-stimulatory domains had superior resistance to Tregs compared to CAR T-cells containing only a CD3 domain (93). Importantly, these findings were observed in the presence of Tregs, demonstrating the immunosuppressive effect of Tregs, and also showing that the domains comprising the CAR itself may affect interactions with the TME.

## Discussion: Strategies to Befriend the TME in CAR T-Cell Therapy

### Altering the Cytokine Milieu

**Figure 3:**
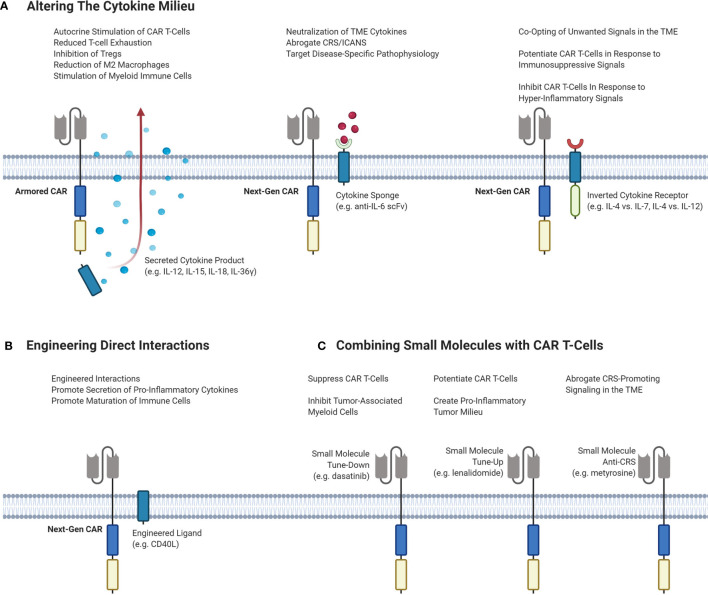
Different strategies of targeting the TME to enhance the efficacy and decrease the toxicity of CAR T-cells. **(A)** Potential CAR T-cell strategies that can modulate the cytokine milieu in the TME. Armored CARs are second-generation CARs that are engineered to secrete a cytokine product of interest to stimulate inflammation and inhibit immunosuppressive cells and signals. Next-Gen CARs can be engineered to neutralize cytokines to dampen CRS/ICANS or to target pathophysiological signaling. Next-Gen CARs can also be engineered to co-express an inverted cytokine receptor that can co-opt various signals in the TME. This can be done to tune-up CAR T-cells in response to immunosuppressive signaling, or to tune-down CAR T-cells in response to hyper-inflammatory signaling to abrogate CRS. **(B)** Engineering CAR T-cells to express a ligand of interest on the T-cell surface to induce interactions or signaling pathways of interest. **(C)** Small molecules can be administered to achieve different effects with CAR T-cells. Small molecules can serve to either tune-up or tune-down CAR T-cells or be used to abrogate CRS-potentiating signaling. CAR, chimeric antigen receptor; Treg, regulatory T-cell; TME, tumor microenvironment; CRS, cytokine release syndrome; ICANS, immune effector cell-associated neurotoxicity syndrome; IL, interleukin; scFv, single-chain variable fragment.

An attractive option to improve responses by altering the TME is by exogeneous cytokine administration. However, early clinical trials of systemic recombinant cytokines such as IL-2, IL-12 and IL-15 demonstrated toxicity at higher doses, limiting efficacy. In the era of genetically engineered cell therapies, CAR T-cells can carry a payload that directly alters the cytokine milieu in the TME without severe systemic effects. Strategies to alter the TME with CAR T-cell therapy are outlined in **Figure 3**.

IL-12 is a main proinflammatory cytokine that stimulates T-cell responses by priming them for IFN-γ production, inducing apoptosis of regulatory T-cells, and increasing CD8^+^ T-cell infiltration (94–97). Furthermore, adoptive transfer of CD8^+^ T-cells engineered to secrete IL-12 enhanced responses in a murine model of melanoma by reprogramming MDSCs (98). CAR T-cells engineered with constitutive or inducible expression of IL-12 have demonstrated responses in murine models of hepatocellular carcinoma (99), ovarian cancer (100), and lymphoma (101). In a murine immunocompetent model of lymphoma, IL-12-secreting CAR T-cells were found to recruit endogenous T-cells and induce epitope spreading (101).

IL-15 is a proinflammatory cytokine important for the memory differentiation and proliferation of T-cells and NK cells (102). In a clinical trial of CD19 CAR T-cells for lymphoma, higher levels of IL-15 were associated with remission (28). CD19 CAR T-cells engineered to constitutively secrete IL-15 demonstrated improved proliferation upon exposure to its cognate antigen, improved CAR T-cell persistence, reduced expression of the PD-1 exhaustion marker, and improved tumor clearance *in vivo* (103).

CAR T-cells engineered with IL-18 have also been developed, with the hypothesis that IL-18 would maintain CAR T-cells in an early effector stage by inducing a T-Bet^high^FoxO1^low^ signature (104). When applying this armored CAR T-cell therapy to a murine model of pancreatic cancer, the authors discovered that this engineered CAR T-cell also converted the immune cell landscape toward acute inflammation by reducing the frequency of M2 macrophages and Tregs in the tumor, leading to an enhanced response and tumor clearance. However, no differences in the frequency or type of MDSCs was seen. On tumor re-challenge, the IL-18 expressing CAR T-cells also demonstrated serial killing capacity with decreased exhaustion markers compared to CAR T-cells without IL-18 (104).

Another approach to enhance responses involves engineering CARs to secrete IL-36γ, a novel group of cytokines belonging to the IL-1 superfamily. In a murine model of lymphoma, CAR T-cells secreting IL-36γ resulted in improved tumor eradication compared to CAR T-cells alone (105). Furthermore, these CAR T-cells activated DCs *in vitro* and increased the percentages of CD86^+^ MHC-II^+^ DCs and macrophages *in vivo*, demonstrating the activation of endogenous antigen-presenting cells (105).

However, with all these cytokine-altering therapies outlined, it is still unknown what effect these approaches will have on CRS and ICANS as they have not been fully investigated in models that are known to recapitulate CRS. In fact, cytokine alterations may in fact worsen toxicity; for example, the IL-36γ secreting CAR T-cells described above induced significant production of IL-6, one of the main cytokines implicated in CRS (105). Therefore, until these cytokine-enhancing therapies are tested in preclinical models of CRS or results are seen in early phase clinical trials, it is as of yet unclear if these approaches will truly enhance the therapeutic potential of CAR T-cell therapy.

One interesting approach to alter the cytokine milieu that is specifically directed at decreasing toxicity is to engineer CAR T-cells that can neutralize certain factors, such as IL-6. Tan et al. developed a CD19 CAR T-cell with a membrane bound scFv targeting IL-6 constitutively expressed on its surface. This construct effectively neutralized macrophage-derived IL-6 without compromising anti-tumor efficacy (106). Of note, reducing IL-6 in the TME may not only help abrogate the symptoms of CRS, but it has the potential to simultaneously limit the effect of TAMs, since TME-derived IL-6 is often implicated in the pathogenesis of many cancers due to its positive feedback loops that promote tumor growth, such as in ovarian cancer, breast cancer, and multiple myeloma (107–110).

A potential strategy that could be explored is engineering CAR T-cells that co-express a chimeric receptor that can co-opt unwanted signals in the TME and redirect them towards favorable effects. For example, low-affinity receptors for IL-6 could be fused to an immunoinhibitory intracellular signaling domain to dampen CAR T-cell function when high IL-6 concentrations lead to binding of this receptor. Indeed, so-called inverted cytokine receptors (ICRs) have been studied to redirect the immunosuppressive of certain cytokines in the TME towards pro-CAR T-cell effects. An IL-4 vs. IL-7 (4/7) ICR comprised of the IL-4 receptor exodomain fused to the IL-7 receptor endodomain resulted in enhanced proliferation and activation of Epstein-Barr virus (EBV)-specific effector T-cells rather than inhibition, ultimately resulting in superior antitumor activity (111). This effect was mediated through activation of the IL-7 endodomain which activated a signal cascade that results in a pro-inflammatory Th1 program (111). This approach has tremendous potential and may be amenable to *in vivo* TME modulation by CAR T-cells, particularly in solid tumors where IL-4 is a major contributor towards immunosuppression and neutralization of IL-4 using monoclonal antibodies or deletion of IL-4-expressing T follicular helper cells has been shown to improve T-cell trafficking to tumors and T-cell-mediated anti-tumor functions (112–115). Indeed, a 4/7 ICR co-expressed in prostate stem cell antigen (PSCA)-directed CAR T-cells reversed immunosuppression and led to enhanced CAR T-cell-mediated cytotoxicity in a mouse model of pancreatic ductal adenocarcinoma (116). These promising results with ICRs have led to further ICR investigations, such as the novel IL-4 vs. IL-21 ICR (4/21) that promotes Th17-like polarization in CAR T-cells in response to IL-4 in the TME that resulted in improved tumor-clearance *in vivo* (117). Whereas the objective of the 4/7 and 4/21 ICR co-expressing CAR T-cells is to potentiate CAR T-cell function by redirecting immunosuppressive signals in the TME into pro-CAR T-cell signals, a similar approach to downregulate CAR T-cell function as a form of negative feedback could also be studied. Such negative-feedback CAR T-cells may serve a dual role of not only potentially protecting against CRS and ICANS, but also mitigating CAR T-cell exhaustion.

Another strategy is that outlined by Sterner et al. and described above, where CAR T-cells are engineered to knockout certain cytokines that help initiate CRS, such as GM-CSF (42). This method appeared to not only reduce CRS but also enhanced responses. An early phase clinical trial combining axi-cel with GM-CSF blockade using lenzilumab is currently ongoing (ZUMA-19, NCT04314843), and the results of this trial may be informative as to wherever or not GM-CSF knockout CAR T-cells should be pursued clinically.

### Engineering Direct Interactions

CD19-directed CAR T-cells engineered to constitutively express CD40 ligand (CD40L, CD145) were demonstrated to have increased tumor clearance in a xenograft model of CD19^+^ lymphoma, increased CAR T-cell proliferation, as well as increased the secretion of pro-inflammatory Th1 cytokines (118). Interestingly, this strategy also resulted in the maturation of monocyte-derived dendritic cells and the secretion of IL-12. However, enthusiasm for this approach is tempered by the fact that in the murine model of CRS described by Giavridis et al., mCD40L engineered CAR T-cells worsened CRS, highlighting again the importance of balancing toxicity and efficacy (35).

### Combining Small Molecules With CAR T-Cells

One of the most straightforward methods of fine-tuning CAR T-cell function and altering the TME is the use of pharmacological molecules. Ibrutinib, a Bruton tyrosine kinase inhibitor, was one of the first molecules to be used in this way given its known immunomodulatory effects on the T-cell compartment in CLL by favoring Th1 immunity (119, 120). Fraietta et al. demonstrated that concurrent ibrutinib therapy improved engraftment and efficacy of CD19 CAR T-cells in murine xenograft models of ALL and CLL (121). In a separate study using a murine mantle cell lymphoma model, Ruella et al. demonstrated that ibrutinib worked synergistically with CD19 CAR T-cells to more effectively clear tumor burden (122). Subsequent clinical trials of concurrent ibrutinib with CD19 CAR T-cells in CLL have demonstrated lower CRS severity with equivalent or better efficacy (123). This highlights that some molecules, such as ibrutinib, may be able to reduce CRS while simultaneously deepening responses.

Recently, considerable work has been done to study how lenalidomide, an immune-modulatory drug, can be used to potentiate CAR T-cells. Indeed several studies have shown that treatment of CAR T-cells with lenalidomide improved CAR T-cell cytokine release and cytotoxicity *in vitro*, and improved tumor clearance in mouse models of multiple myeloma and lymphoma (124–126). In another murine model of CRS, Ruella et al. demonstrated that ruxolitinib, a JAK/STAT pathway inhibitor with inflammatory cytokine dampening properties in myelofibrosis and graft-versus-host disease, attenuated CRS without impairing efficacy (127).

Dasatinib, a tyrosine kinase inhibitor, has been studied by Mestermann et al. as a way to limit toxicity of CAR T-cells (128). They showed that dasatinib immediately induces a function-off state in CD4^+^ and CD8^+^ CAR T-cells that is dose dependent, reversible, and does not affect T-cell viability. In mice engrafted with CD19^+^ Raji lymphoma cells, those infused with CD19-directed CAR T-cells rapidly developed CRS, and administration of dasatinib significantly decreased levels of IFN-γ, GM-CSF, IL-2, and TNF-α and protected mice from fatal CRS. In addition to exerting precise control over CAR T-cells, dasatinib can also directly inhibit tumor-associated myeloid cells suggesting that an additional benefit of dasatinib (129).

Another example of the use of a small molecule is the use of α-methyltyrosine (metyrosine, MTR) to inhibit catecholamine synthesis as described by Ferrari et al. (44). They showed that treatment of CD19 CAR T-cells incubated with CD19^+^ Raji cells with MTR reduced catecholamine and pro-inflammatory cytokine levels *in vitro* (44). They further showed that pre-treatment of tumor-bearing mice with MTR prior to CAR T-cell infusion decreased plasma adrenaline and noradrenaline concentrations, reduced human CAR T-cell-derived IFN-γ and TNF-α, and reduced mouse-derived IL-6 and CXCL1 (44). MTR did not affect CAR T-cell efficacy, as neither tumor clearance nor CAR T-cell expansion was abated, thus supporting the rationale for the use of MTR to reduce systemic pro-inflammatory signaling associated with CRS without compromising anti-tumor function.

## Future Perspectives

CAR T-cell immunotherapy offers new hope for patients with cancer and is at the forefront of a paradigm shift in cancer therapy. However, this has led to novel immune-related toxicities which we are just starting to understand from a pathophysiologic perspective. Through this, we have seen that the TME plays a dual role in both driving toxicity and hampering efficacy. This knowledge has spurred the development of newer generations of TME-modulating CAR T-cells and/or combinations with CAR T-cells, with a hope to simultaneously increase efficacy and decrease toxicity. Several of the approaches we have discussed show promise in preclinical models; however, clinical trials are necessary due to a lack of testing in preclinical models that faithfully recapitulate both tumor and TME. Without the use of immunocompetent preclinical models that do this with high fidelity, it will be difficult to clearly identify the most optimal method until more of these regimens reach clinical trials and transitional work is performed. Furthermore, given that the TME is not identical between different cancers it is likely that some strategies may be more effective in certain malignancies than others. Therefore, the translational work from these clinical trials and the development of high-fidelity immunocompetent disease models will help guide further studies, especially those targeting a variety of solid tumors.

## Author Contributions

LL, LW, and KH conceptualized, wrote, and edited the manuscript. LL and LH designed the figures. All authors contributed to the article and approved the submitted version.

## Funding

KH receives funding from the British Columbia Cancer Foundation and the VGH and UBC Hospital Foundation that supported this review.

## Conflict of Interest

KH has served on ad hoc advisory boards for Gilead/Kite, Novartis, and BMS.

The remaining authors declare that the research was conducted in the absence of any commercial or financial relationships that could be construed as a potential conflict of interest.

## Abbreviations

CAR, chimeric antigen receptor; Treg, regulatory T-cell; TME, tumor microenvironment; CRS, cytokine release syndrome; ICANS, immune effector cell-associated neurotoxicity syndrome; IL, interleukin; scFv, single-chain variable fragment.
